# The Reliability, Validity and Normative Scores of the Bene-Anthony Family Relations Test for Use With Arab Children

**DOI:** 10.3389/fpsyg.2021.548493

**Published:** 2021-01-27

**Authors:** Abdrabo Soliman, Abdel-Salam G. Abdel-Salam, Mervat Ahmed

**Affiliations:** ^1^Department of Social Sciences, Qatar University, Doha, Qatar; ^2^Department of Mathematics, Statistics and Physics, College of Arts and Sciences, Qatar University, Doha, Qatar; ^3^The Family Consulting Center, Qatar Foundation, Doha, Qatar

**Keywords:** family dynamics, Arab children, Bene-Anthony Family Relations Test, normative scores, reliability, validity

## Abstract

Background: The Bene-Anthony Family Relations Test (BAFRT) is one of the most widely used measures of family dynamics seen from a child’s perspective. However, the most common issue surrounding this test is the lack of accurate normative scores for use with non-white ethnic groups. The purpose of this study was to examine the BAFRT’s reliability and validity for use with Arab children, as well as to provide normative data for this group. Methods: The BAFRT was translated into Arabic and back-translated to ensure accuracy. The test was administered to a cohort of 394 Arab children, consisting of both cognitively normal children (*n* = 269) and children diagnosed with a psychological disorder (*n* = 125), all aged 5–8 years old. Test-retest reliability was assessed using a sub-set of children and validity was tested against clinical status as well as CBCL and SDQ measures. Normative measures were calculated after examining the impact of influencing variables such as age and gender. Results: Statistical analyses showed that in our cohort of Arab children the BAFRT has good test-retest reliability, correlates well with measures of emotional and behavioral adjustment, and discriminates accurately between clinical and non-clinical children. Age, gender, and clinical status all significantly impacted upon BAFRT scores and therefore normative values are presented from our cohort when considering these variables. Conclusion: The normative scores we present will provide researchers and clinicians an appropriate reference point for the comparison of scores from Arab children and a starting point for future research into this area.

## Introduction

Children’s perspectives on family dynamics is an important insight to collect accurately for both family psychology research and clinical practice. Relationships with family members can strongly influence a child’s behavior, personality and play an important role in childhood mental health (Dunn et al., 1994; Garmezy and Masten, 1994; Shaw et al., 1994). It is imperative to try to understand these relationships from the child’s perspective but many measurement tools instead rely on adult observations of the child’s relationships. One measure that does attempt to assess these relationships from a child’s perspective is the Bene-Anthony Family Relations Test (BAFRT; Bene and Anthony, 1957).

The BAFRT is a forced-choice questionnaire presented to children in the form of a game. It is designed to provide insights and enable the evaluation of a child’s attitude to and relationships with parents and other family members using projective techniques. Originally published in 1957, the test has since been widely adopted as a useful tool helped by the fact that it involves a relatively simple task that does not require speech, meaning very young children and those who find it difficult to express themselves verbally can understand and complete the BAFRT with ease. The test consists of several figures depicting ambiguous people of various ages but without facial features. The children select one figure to represent each member of their family, including one figure for themselves. Each figure is attached to a post box through which statements can be posted that the child associates with that figure. Depending on the age of the child, statements vary in valence (positive/negative), strength (mild/strong), and direction (outgoing from child/incoming to child). A final figure “nobody” is also added to the group to whom any statements not assigned to a family member can be given.

The test was designed to provide an insight and a way of measuring the emotional attitudes of children to their family members and also the children’s views of family member attitudes toward them (Frost, 1969). From the simple task, a number of feelings and mechanisms can be calculated or deduced, including; feelings of importance and ambivalence toward family members, defense mechanisms, inhibition/disinhibition and total outgoing and incoming feelings (Parkin, 2001). Although the BAFRT has been shown to have a positive diagnostic ability with certain groups, the main value of the BAFRT is as an evaluation technique to provide individual insight into family dynamics, behaviors, and feelings. When questioned, intern-psychologists who used the BAFRT in psychotherapeutic treatment felt that the test deepened their understanding of the nature and quality of their clients family dynamics and that relationships were revealed to be much more complex after the application of the test, with negative relationships particularly clarified (Brand, 1996). This demonstrates the general value of the BAFRT in a clinical setting especially for the evaluation of a dysfunctional family environment.

Although the test results can be evaluated in depth at a case study level, the organized structure of the test does still enable quantitative scores to be calculated which can be compared to normative values. Over the past 60 years the FRT has been used across different ages, socio-economic settings and genders. The test has also been used to compare children from populations that differ in some way, examining differences resulting from various clinical diagnoses, socioeconomic settings, childhood experiences, and others (Frost, 1969; Matějček et al., 1978; Turner, 1982; Rosen and Brigham, 1984; Moore and Nystul, 2011). Therefore, values can be found for comparison across many different populations of children. However, despite its frequent and wide use, one of the most common issues surrounding this test is the lack of accurate normative scores for use with non-white ethnic groups; a limitation that needs addressing in order to compare results and increase the accuracy and validity of normative values across ethnicities and cultures.

The BAFRT has been repeatedly tested, validated, and demonstrated high clinical and research utility in Western populations for which normative scores are readily available (e.g., Frost, 1969; Flaemig and Woerner, 1977). However, it is important to assess the applicability to other cultures and ethnicities. For example, a recent assessment of the BAFRT in an Indian setting revealed the poor value of the test with Indian children due to a poor level of identification with the BAFRT figure images used (Ranjan et al., 2017). This information is of high value for practitioners who can instead now select a more culturally appropriate and beneficial test for both the practitioner and the child. Results such as those obtained by Ranjan et al. (2017) highlight the importance of assessing the value of the BAFRT across countries, ethnicities, and cultures and the importance of culturally appropriate normative values. Ensuring that an individual’s performance is compared to the appropriate standard helps to ensure that inaccurate characterization of test scores, over- and under-pathologizing, or poor prediction of a given characteristic can all be avoided. Demographically appropriate norms, such as those that are suitable for the child’s age, education, sex, and race/ethnicity, can improve specificity and sensitivity of a clinical test.

Here we start to address this limitation by assessing the performance of the BAFRT in a cohort of solely Arab children. Family dynamics in Arab countries are faced with many challenges, both those common to other cultures and those more unique to the Arab setting, that can negatively affect the relationship between family members and threaten children’s overall social and psychological wellbeing. These challenges include but are not limited to living conditions, women’s work, marginalization of the role of motherhood and marital relations, the high rates of divorce in recent years, as well as globalization, the media, and modern means of communication. Unstable family dynamics can disrupt children’s psychosocial development, hence children of disrupted families subjected to parental problematic issues are classified as an at-risk population. This study firstly aimed to examine the reliability of the BAFRT in this cohort, and also aimed to assess its validity by determining both its correlation to measures of behavioral and emotional adjustment and its ability to differentiate between cognitively normal children and those clinically diagnosed with psychological disorders. It has been previously found that a child’s gender and age impacts upon their feelings toward family members and that this is reflected in the scores obtained from the BAFRT (Rosen and Brigham, 1984). Therefore, the impact of age and gender as well as clinical status of the child upon BAFRT scores was also examined here in order to inform the calculation of normative values from our cohort. To our knowledge this is the first study that has used the BAFRT in an Arab population and it is hoped that the results and resulting normative scores will provide researchers and practitioners with a valid and reliable reference point of Arab children’s perception of family relationships.

## Materials and Methods

### Participants

In total, 394 Arab children, ranging in age from 5 to 8 years old (Mean = 7.4 years, SD = 1.47), spanning all socio-economic classes, participated in this study. Children were recruited to the study from 11 centers located in Tanta City, Egypt (3 public schools and 8 outpatient psychological service centers), and via word of mouth. For inclusion to the study, children were required to have an IQ above 90, as measured by the Stanford-Binet Intelligence Scales Fifth Edition (Roid and Pomplun, 2012), and all children were living with both parents. All children and their families spoke Arabic and were resident in either Egypt (*n* = 279) or Qatar (*n* = 115). Only one child per family was included in the study; no siblings participated.

As we wanted to assess the influence of age, gender and cognitive health upon BAFRT scores, children were assigned to groups based upon these variables for analysis purposes. Age was split into four age groups; 5, 6, 7 and 8 years old. Children were assigned to the “cognitively normal” group dependent upon the absence of a current psychological disorder and a clear history of mental or developmental disorders. Inclusion in the “clinical” group was dependent upon the diagnosis of any psychological disorder. The N-number, mean, SD, age and gender split for each group is given in Table 1. Limited to the exploratory nature and the sample size in the current study, and due to the significant variations in the dependent variables based on diagnosis (normal and abnormal), gender, and target family members we did not use complex statical modeling. Based on recommendations for SEM there should be at least 5 or 10 observations per estimated parameter (e.g., Bentler and Chou, 1987). Therefore, none of samples (boys, girls, normal or abnormal) would be satisfactory to fit the models. Therefore, we examined only convergent validity and tested the predictive ability of the individual BAFRT variables for the discrimination of cognitively healthy and clinical groups.

**TABLE 1:** Descriptive statistics for children across the whole cohort.

**Groups**	**Age Cohorts**	***N***	**% Boys**	**Mean**	**SD**
Clinical group	5 years group	48	29	4.55	0.26
	6 years group	24	24	5.53	0.29
	7 years group	34	24	6.51	0.24
	8 years group	19	22	7.43	0.26
	Total	125	36	5.71	1.11
Cognitively normal group	5 years group	43	15	4.60	0.29
	6 years group	78	28	5.53	0.30
	7 years group	62	26	6.42	0.30
	8 years group	86	31	7.45	0.26
	Total	269	34	6.20	1.07
Whole cohort	5 years group	91	20	4.57	0.28
	6 years group	102	27	5.53	0.30
	7 years group	96	25	6.45	0.28
	8 years group	105	28	7.45	0.26
	Total	394	35	6.04	1.10

### Measures

#### The Bene-Anthony Family Relations Test (BAFRT)

The BAFRT (Bene and Anthony, 1957) is a clinical tool that enables a quick, objective and quantitative insight into a child’s relationship with their family members. In our study, figures were presented to the children as drawings attached to post-boxes and the children selected a figure to represent each member of their family. There are two versions of the BAFRT dependent on the age of the child being tested. Typically, the younger version (Bene and Anthony, 1985) is used for children 8 years and younger. Therefore, this version was selected for use in our study. This version of the BAFRT consists of 40 statements that vary in valence (positive/negative) and direction (outgoing from child/incoming to child). The statements are grouped into five categories for scoring purposes: (1) positive outgoing feelings, (2) negative outgoing feelings, (3) positive incoming feelings, (4) incoming negative feelings, (5) dependent feelings. The BAFRT was administered to all 394 children. The test was conducted in the child’s school or clinical center by either a research assistant or a clinical psychologist, with one child being tested at a time. As children were assessed by more than one examiner, inter-rater reliability between raters was examined and determined to be reliable using the Intraclass correlation coefficient by a two-way random effect model (ICC > 0.9). The BAFRT was translated into Arabic and then back-translated to check the accuracy of the translation.

#### The Child Behavior Checklist (CBCL)

The Child Behavior Checklist (CBCL; (Achenbach and Edelbrock, 1983) is a 118-item scale that examines multiple behavioral and emotional issues. This study utilized the complete scale in order to assess multiple factors such as internalizing and externalizing and to provide an overall interpretation of the severity of any impairment. The CBCL was completed for a subset of 68 children, by the child’s father, mother and teacher, who all completed the measure in their own homes. The CBCL was already available in Arabic.

#### Prosocial Behavior Strengths and Difficulties Questionnaire (SDQ)

The Prosocial Behavior Strengths and Difficulties Questionnaire (SDQ; Goodman, 1997) is a 25-item screening instrument consisting of five sub-scales that each measure children’s strengths and difficulties in a different domain: Emotional Symptoms, Conduct Problems, Hyperactivity/Inattention, Peer Relationships Problems, and Prosocial Behavior. For the purpose of the current study, only the Prosocial Behavior sub-scale was used. Answers were rated on a three-point Likert scale (0 = not true at all to 2 = definitely true). The SDQ was completed for the same subset of 68 children as the CBCL, by the child’s teacher who completed the measure in their own home. The SDQ was already available in Arabic.

### Ethics

Informed consent was obtained from all children via the schools and clinical centers. Participants parents signed an informed consent form. Ethical approval for this study was obtained from the Ethical Committee in the Department of Psychology, Tanta University, Egypt.

### Statistical Analysis

Statistical analysis was conducted using IBM SPSS Statistics software (version 26, SPSS Inc., Chicago, IL, United States) and MedCalc (Version 19.1.3, MedCalc Software Ltd., Belgium). The analysis consisted of three different phases: reliability analysis (phase 1), validity analysis (phase 2), and normative data generation (phase 3). In phase 1, Pearson’s correlation coefficient was used to examine the test-retest reliability for the 27 BAFRT Variables. Phase 2 again utilized Pearson’s correlation coefficient to examine the relationship between BAFRT, CBCL, and SDQ scores. *T*-tests and Receiver Operating Characteristic (ROC) analysis was then used to establish how well each of the 27 BAFRT Variables was able to differentiate between cognitively normal and clinical groups; the area under the curve (AUC), sensitivity and specificity are reported for each BAFRT variable. Phase 3 firstly involved two-way analysis of variance (ANOVA), several one-way ANOVA and independent sample *t*-tests to examine the differences in the 27 BAFRT variables between genders, cognitively normal and clinical groups and also across age groups. Finally, normative data was generated and presented as a simple mean and standard deviation calculations. This approach ensured the figures presented are simple to understand in order to facilitate interpretation and future comparison of results. Normative scores were provided for each sub-group of children for which a variable was found to impact BAFRT scores in our earlier two-way ANOVA, one-way ANOVA and t-tests analyses.

## Results

### Phase 1: Reliability Analysis

#### Test-Retest Reliability

In order to examine test reliability and internal consistency, the BAFRT was administered twice to a sub-sample of 68 cognitively normal children (26 boys, 42 girls; mean age = 6.28 years, SD = 3.012). There was a three-week time interval between the two measurements (mean interval = 18.22 days, SD = 2.01). Pearson’s correlation coefficient was computed for each BAFRT variable to assess the relationship between the two tests. A significant positive correlation was found for every variable of the BAFRT (*p* < 0.01) indicating that the results remain stable and reliable, over time; the results are displayed in Supplementary Table S1.

#### Replication of Test-Retest Reliability

In order to replicate the test re-test reliability and internal consistency, the BAFRT was administered to an independent sub-sample of 64 normal children (28 boys, 38 girls; mean age = 6.53 years, SD = 2.31). As before, there was a three-week time interval between the two measurements (mean interval = 18.22 days, SD = 2.01). Pearson’s correlation coefficient was computed for each BAFRT variable to assess the relationship between the two tests and enable a comparison to the previous analysis. Although overall slightly more moderate correlations were reported in this analysis, all correlations were highly significant (*p* < 0.01), replicating the results of the original test-retest analysis. These results are displayed in Supplementary Table S2.

### Phase 2: Convergent Validity Analysis

#### Correlation Between BAFRT Variables and CBCL (Father, Mother, and Teacher) and SDQ Prosocial Behavior Scores

To examine the convergent validity of the BAFRT, the Pearson’s correlation coefficient was calculated to examine the relationship between each of the 27 BAFRT variables and the total score of CBCL (from the father, mother, and teacher) and the prosocial behavior scale from the SDQ (teacher version). The measures where administered to a sub-sample of 68 children (mean age = 7.01, SD = 1.73;% boys = 38%). Both the CBCL and the SDQ scores correlated significantly with all of the BAFRT variables (*p* < 0.01). Variables within the BAFRT negative outgoing, negative, and the dependency feelings categories were all significantly positively correlated with CBCL and SDQ scores (*p* < 0.01). BAFRT variables in both the positive incoming and outgoing feeling categories were significantly negatively correlated with CBCL and SDQ scores (*p* < 0.01). These results are displayed in Supplementary Table S3.

#### Between-Group Differences; Cognitively Healthy and Clinical Groups

In order to test if the BAFRT scores differed between children in the cognitively healthy and clinical groups, an independent sample *t*-test for a two-sample was conducted utilizing data across the whole cohort (total *N* = 394; cognitively healthy group *N* = 269, clinical group *N* = 125). The normality and homogeneity assumptions for using the parametric approach were investigated using Shapiro–Wilk test and Levene’s test, respectively, for more details see Verma and Abdel-Salam (2019). However, the normality could be assumed based on the Central Limit Theorem for a large sample size (*n* > 30). Consequently, the two-sample *t*-test utilized and showed a statistically significant difference between groups for each of the 27 BAFRT variables (*p* < 0.01), indicating that the BAFRT variables are sensitive to the clinical status of Arab children. These results are displayed in Supplementary Table S4.

#### ROC Analysis for the Discrimination of Clinical and Cognitively Normal Children

ROC and AUC statistics were used to determine the predictive ability of the individual BAFRT variables for the discrimination of cognitively healthy and clinical groups. Table 2 displays the results of the ROC analysis and Supplementary Figure S1 displays the ROC curves for each variable. The AUC for every variable was far higher than chance (0.5), even when including the lower end of the confidence interval. The variable with the highest accuracy for discriminating between groups was dependency feelings, father (AUC = 96.7%).

**TABLE 2:** ROC and AUC statistics for each of the 27 BAFRT variables for the discrimination between cognitively healthy and clinical groups.

**Variables\Parameters**	**Cut-off value**	**Sensitivity (%)**	**Specificity (%)**	**AUC (95% CI)**	***p***
**Negative outgoing feelings**					
Mother	≤3.85	81.78	88.00	0.922 (0.891 to 0.947)	<0.01
Father	≤3.08	95.91	60.00	0.890 (0.855 to 0.919)	<0.01
Self	≤3.07	85.50	85.20	0.909 (0.876 to 0.935)	<0.01
Sibling	≤3.08	82.53	83.20	0.901 (0.878 to 0.937)	<0.01
Friend	≤3.08	86.62	83.20	0.925 (0.894 to 0.949)	<0.01
Nobody	≤3.08	86.25	81.60	0.911 (0.877 to 0.936)	<0.01
**Negative incoming feelings**					
Mother	≤3.08	83.64	86.40	0.923 (0.892 to 0.947)	<0.01
Father	≤3.08	83.64	81.60	0.884 (0.848 to 0.914)	<0.01
Self	≤3.08	82.90	84.00	0.908 (0.875 to 0.934)	<0.01
Sibling	≤3.08	83.64	84.80	0.908 (0.875 to 0.934)	<0.01
Friend	≤3.08	83.27	85.60	0.905 (0.872 to 0.932)	<0.01
Nobody	≤3.08	84.01	80.00	0.901 (0.867 to 0.928)	<0.01
**Positive outgoing feeling**					
Mother	>2.31	90.33	63.20	0.850 (0.811 to 0.884)	<0.01
Father	>3.08	57.62	84.80	0.771 (0.727 to 0.812)	<0.01
Self	>3.08	57.25	86.40	0.793 (0.749 to 0.832)	<0.01
Sibling	>3.08	52.42	87.20	0.765 (0.720 to 0.806)	<0.01
Friend	>3.08	59.85	85.60	0.804 (0.761 to 0.842)	<0.01
Nobody	>3.08	57.99	84.80	0.783 (0.739 to 0.823)	<0.01
**Positive incoming feelings**					
Mother	>3.08	58.36	80.80	0.765 (0.720 to 0.806)	<0.01
Father	>2.31	88.48	60.80	0.833 (0.793 to 0.869)	<0.01
Self	>3.08	53.53	80.80	0.748 (0.702 to 0.790)	<0.01
Sibling	>3.08	54.65	83.20	0.753 (0.708 to 0.795)	<0.01
Friend	>2.31	84.01	61.60	0.786 (0.742 to 0.825)	<0.01
Nobody	>3.08	56.88	86.40	0.808 (0.765 to 0.845)	<0.01
**Dependency feelings**					
Mother	≤2.31	92.94	84.51	0.948 (0.922 to 0.968)	<0.01
Father	≤3.08	94.05	88.80	0.967 (0.944 to 0.982)	<0.01
Sibling	≤2.31	85.50	79.20	0.902 (0.868 to 0.929	<0.01

### Phase 3: Normative Data Generation

Variables known to influence BAFRT scores, age and gender, were firstly assessed to identify if they had a significant effect upon BAFRT scores in this cohort in order to inform the subsequent calculation of normative values.

#### Investigating the Influence of Age Upon BAFRT Scores

To assess the influence of age upon BAFRT scores, a one-way ANOVA was conducted between age groups across the whole cohort (*N* = 394). Also, the normality and homogeneity assumptions were tested. All but one variable was significantly influenced by age (*p* < 0.05). The variable not significantly influenced by age was positive incoming feelings toward self (*p* > 0.05). The ANOVA results are displayed in Supplementary Table S5.

In order to determine if age significantly influences BAFRT scores for healthy and clinical children using two-way ANOVA, considering the interaction between age and healthy/clinical children (age × group) but the results showed that the interaction was not statistically significant (*p* > 0.05). Hence, the main-effects tested independently using one-way ANOVA for each group. The results from the clinical group (*N* = 125) show that age has a significant impact on all variables in the dependency feelings category and 4/5 variables in both the negative outgoing and incoming feelings categories (*p* < 0.05). Age did not significantly influence the BAFRT scores in either the positive incoming and outgoing feelings categories (*p* > 0.05). ANOVA results for the clinical group are displayed in Supplementary Table S6. Results from the cognitively healthy group (*N* = 269) show that age has a significant impact upon all variables in the negative outgoing, negative incoming and dependency feelings category (*p* < 0.05). From the positive incoming and outgoing feelings categories, only one variable was significantly influenced by age; positive outgoing feelings assigned to a friend (*p* < 0.01). These results are displayed in Supplementary Table S7.

#### Investigating the Influence of Gender Upon BAFRT Scores

To assess the influence of gender upon BAFRT scores, an independent samples t-test was conducted between genders, across the whole cohort (*N* = 394). Also, the normality and homogeneity assumptions were tested using Shapiro–Wilk test and Levene’s test, respectively. Gender was found to have a significant influence on all scores in the negative outgoing, negative incoming and dependency feelings categories (*p* < 0.01). Scores within the positive incoming and outgoing feelings categories were not significantly influenced by gender (*p* > 0.05). The results from this analysis are presented in Supplementary Table S8.

In order to determine if gender significantly influences BAFRT scores for cognitively healthy and clinical children, a two-way ANOVA was utilized for testing the interaction between gender and healthy/clinical children (gender × group) as well as the two-main effects. The results showed that the interaction was not statistically significant (*p* > 0.05). Hence, an independent samples t-test was conducted for each group independently. Gender was found to have a significant influence on all scores in the negative outgoing, negative incoming and dependency feelings categories for both clinical and cognitively healthy groups of children (*p* < 0.05). Also, significantly affected by gender for clinical children was positive outgoing feelings (*p* < 0.05). This variable was not significant for the cognitively healthy group but interestingly, positive incoming feelings was significantly affected for this group (*p* < 0.05). None of the other variables were significant (*p* > 0.05). The results from the clinical group (*N* = 125) and cognitively healthy group (*N* = 269) are displayed in Supplementary Tables S9 and S10, respectively.

#### Calculation of Normative Values

We sought to calculate quick and simple reference values that could have immediate real-world applications for researchers and practitioners who may use the BAFRT with Arab children. Therefore, normative values were calculated using simple mean and standard deviation calculations that are straightforward to interpret and compare to. As our previous analyses had found age, gender and clinical status to have an overall significant influence upon BAFRT scores, we considered it imperative that these results were presented separately for each sub-group (Table 3).

**TABLE 3:** Normative values of the BAFRT in an Arab cohort; presented as means and standard deviation by age groups, gender, and clinical status.

**Age (Total *N* = 394)**																	
	
		**5 Years Old (*n* = 91)**				**6 Years Old (*n* = 102)**				**7 Years Old (*n* = 96)**				**8 Years Old (*n* = 105)**			
		**Diagnosis**				**Diagnosis**				**Diagnosis**				**Diagnosis**			
		**Abnormal (*n* = 48)**		**Normal (*n* = 43)**		**Abnormal (*n* = 24)**		**Normal (*n* = 78)**		**Abnormal **(*n* = ** 34)**		**Normal **(*n* = ** 62)**		**Abnormal **(*n* = ** 19)**		**Normal **(*n* = ** 86)**	
		**Gender**		**Gender**		**Gender**		**Gender**		**Gender**		**Gender**		**Gender**		**Gender**	
		**Boys **(*n* = 13)****	**Girls **(*n* = 35)****	**Boys **(*n* = 14)****	**Girls **(*n* = 29)****	**Boys **(*n* = 11)****	**Girls **(*n* = 13)****	**Boys **(*n* = 26)****	**Girls **(*n* = 52)****	**Boys **(*n* = ** 11)**	**Girls **(*n* = ** 23)**	**Boys **(*n* = ** 24)**	**Girls **(*n* = ** 38)**	**Boys **(*n* = ** 10)**	**Girls **(*n* = ** 9)**	**Boys **(*n* = ** 29)**	**Girls **(*n* = ** 57)**
**Dependency feelings**																	
Mother	M	4.44	3.72	2.42	1.70	3.85	4.21	1.66	1.73	5.52	4.59	3.21	2.57	5.15	4.28	3.00	2.53
	SD	0.46	0.44	0.41	0.56	0.60	0.60	0.64	0.93	0.46	0.43	0.59	0.55	0.37	0.41	0.52	0.50
Father	M	3.61	2.93	1.71	1.01	3.01	3.38	0.80	1.08	4.62	3.65	2.44	1.68	4.54	3.51	2.31	1.76
	SD	0.58	0.49	0.62	0.69	0.73	0.92	0.60	0.88	0.48	0.58	0.54	0.69	0.43	0.41	0.50	0.71
Sibling	M	3.61	3.19	1.60	1.06	2.80	2.90	1.10	1.07	4.55	3.71	2.44	1.88	4.47	3.68	2.60	1.35
	SD	0.79	0.88	0.77	0.81	0.93	1.00	0.76	0.99	0.94	0.89	1.01	0.83	0.61	1.07	0.91	0.97
**Negative outgoing feelings**																	
Mother	M	5.09	4.57	3.74	2.81	4.69	4.26	2.99	2.19	5.03	4.12	3.05	2.11	4.54	3.94	2.58	2.09
	SD	1.11	1.02	1.20	0.95	0.64	0.74	0.96	0.80	0.86	1.00	0.92	0.87	0.56	0.71	0.88	0.83
Father	M	5.03	4.92	3.79	2.76	4.55	3.97	2.75	2.22	4.20	3.31	3.08	2.19	4.85	3.94	2.79	2.19
	SD	0.74	0.90	0.77	1.04	1.00	0.88	0.98	0.84	0.93	1.02	0.88	0.96	0.63	0.81	0.95	0.86
Self	M	5.56	4.59	4.07	2.84	4.62	4.03	3.11	2.15	4.69	3.92	3.02	2.41	5.00	4.02	2.58	2.23
	SD	0.95	1.03	1.06	0.83	0.91	0.71	0.77	0.75	0.80	1.08	0.72	0.88	0.74	1.00	0.86	0.93
Sibling	M	5.38	4.64	3.90	2.55	5.31	4.09	2.78	2.27	4.89	3.71	3.11	2.11	4.46	3.51	2.66	1.97
	SD	0.83	0.75	1.15	1.27	0.72	0.58	1.17	0.91	0.62	0.94	1.00	0.94	0.70	0.87	1.00	0.82
Friend	M	5.09	4.51	3.79	2.89	5.04	4.20	2.87	2.16	4.62	3.68	3.05	2.21	4.92	3.51	2.60	1.93
	SD	0.80	0.72	1.11	0.96	0.79	0.92	0.80	0.84	0.68	0.83	0.95	1.00	0.90	1.09	0.97	0.92
Nobody	M	5.27	4.59	3.90	2.71	4.69	3.91	2.93	2.15	4.89	3.68	2.86	2.03	4.23	4.02	2.84	2.11
	SD	1.03	0.82	1.18	0.81	1.00	1.02	0.82	0.91	0.62	0.98	0.83	0.95	0.90	0.75	1.01	0.89
**Negative incoming feelings**																	
Mother	M	5.44	4.55	3.85	2.87	5.03	4.26	3.02	2.09	4.76	4.08	2.86	2.01	4.85	4.02	2.81	2.05
	SD	1.06	0.90	1.17	0.87	0.52	0.81	1.11	0.78	0.75	0.82	1.00	1.05	0.52	0.84	0.95	0.92
Father	M	5.15	4.84	3.57	2.73	4.41	3.32	2.72	2.24	4.90	3.95	2.98	2.07	4.54	3.34	3.08	2.15
	SD	1.10	0.94	0.98	1.02	0.85	0.91	0.90	0.94	0.38	0.93	0.89	0.84	0.76	1.02	0.82	0.92
Self	M	5.38	4.57	3.74	2.79	4.34	4.03	2.72	2.13	4.83	3.78	2.92	2.11	4.92	3.42	2.73	2.04
	SD	0.62	0.93	0.67	0.91	0.62	1.09	0.82	0.77	0.91	0.87	0.85	0.91	0.64	1.09	0.86	0.89
Sibling	M	5.32	4.70	3.85	2.87	4.83	4.32	2.84	2.25	4.76	3.68	2.73	2.21	4.31	4.19	2.76	1.99
	SD	0.58	0.78	1.04	1.05	0.69	0.50	0.84	0.95	1.13	1.13	1.04	1.06	0.82	0.56	0.98	0.82
Friend	M	5.50	4.64	3.63	2.76	4.69	4.09	2.87	1.94	4.96	3.62	2.89	2.21	4.85	4.02	2.71	2.01
	SD	0.76	0.88	0.82	0.67	0.54	0.66	0.96	0.92	0.93	0.63	0.86	0.97	0.81	1.00	1.00	0.81
Nobody	M	5.21	4.46	3.52	2.55	4.69	3.97	2.67	2.19	4.55	3.75	3.11	2.03	4.62	3.93	2.58	2.13
	SD	1.30	0.96	1.08	1.09	1.21	0.82	0.85	0.93	0.87	0.78	0.80	0.88	0.72	1.12	0.72	0.85
**Positive outgoing feeling**																	
Mother	M	2.43	2.73	3.36	3.66	2.38	2.43	3.61	3.58	2.87	2.81	3.40	3.48	2.46	2.65	3.74	3.75
	SD	0.88	0.92	0.57	0.81	0.64	0.82	0.92	0.85	1.09	0.82	1.01	1.14	1.14	1.03	0.91	0.90
Father	M	2.25	2.66	3.52	3.64	2.17	2.61	3.73	3.60	2.31	2.68	3.56	3.42	2.31	2.22	3.93	3.97
	SD	0.86	0.90	0.84	0.96	0.83	0.80	0.77	0.90	0.60	0.77	0.93	0.79	1.09	0.71	0.80	0.83
Self	M	3.08	2.64	3.47	3.64	2.66	2.67	3.49	3.51	2.38	2.88	3.66	3.50	2.08	2.14	3.48	3.65
	SD	0.94	0.90	1.04	0.82	0.87	0.87	0.76	0.96	0.73	0.96	0.94	1.11	0.73	1.20	0.79	0.93
Sibling	M	2.67	2.53	2.86	3.74	2.59	2.61	3.70	3.46	2.66	2.88	3.21	3.61	2.23	2.57	3.85	3.67
	SD	0.97	0.98	0.98	0.87	1.05	0.74	0.92	0.97	0.80	1.02	0.90	1.06	0.67	1.09	1.01	0.91
Friend	M	2.67	2.46	3.63	3.64	2.17	2.13	4.06	3.52	2.52	2.64	3.34	3.36	2.08	2.99	3.53	3.79
	SD	0.81	1.09	0.64	0.96	1.02	0.95	0.94	1.00	0.70	1.01	0.87	0.95	0.89	0.98	0.98	0.97
Nobody	M	2.61	2.44	3.24	3.56	2.24	2.61	3.88	3.72	2.38	2.44	3.43	3.50	2.39	3.08	3.80	3.84
	SD	1.02	0.73	1.21	0.97	0.80	0.97	0.74	0.95	1.00	0.92	0.99	0.87	0.99	0.67	0.79	1.00
**Positive incoming feelings**																	
Mother	M	2.55	2.46	3.46	3.80	2.03	2.55	3.76	3.77	2.24	2.61	3.69	3.53	2.62	2.74	3.90	3.74
	SD	0.73	0.61	0.72	1.03	0.52	0.91	0.90	0.89	0.73	1.06	0.56	0.87	0.83	0.87	0.85	0.83
Father	M	2.78	2.71	3.14	3.82	2.80	2.67	3.97	3.76	2.52	2.75	3.40	3.42	2.62	2.74	3.58	3.81
	SD	0.74	0.96	0.77	1.27	0.62	0.74	0.71	0.96	0.78	0.95	1.01	1.07	0.97	0.56	0.66	0.90
Self	M	2.31	2.55	3.79	3.58	2.59	2.49	3.76	3.39	2.66	2.91	3.46	3.67	2.00	2.65	3.56	3.77
	SD	1.13	0.91	0.88	0.69	0.62	0.71	0.82	0.92	0.72	0.90	0.96	1.14	1.04	0.68	0.93	0.79
Sibling	M	2.84	2.51	3.68	3.58	2.66	2.19	3.88	3.39	2.31	2.68	3.34	3.30	2.39	2.99	3.58	3.69
	SD	0.91	0.75	0.86	0.99	0.63	0.76	0.91	1.08	1.03	0.80	1.03	0.91	0.77	1.12	0.95	1.00
Friend	M	2.72	2.68	3.41	3.77	2.38	2.25	3.67	3.73	2.38	2.68	3.66	3.61	2.23	2.22	3.72	3.88
	SD	0.81	1.09	0.84	1.05	0.80	0.58	1.05	0.92	0.54	0.95	1.09	0.96	0.99	0.46	0.87	1.10
Nobody	M	2.72	2.35	3.08	3.95	2.59	2.78	3.73	3.80	2.52	2.64	3.24	3.42	2.70	2.65	3.58	3.84
	SD	0.74	0.99	0.74	1.14	0.99	0.86	0.94	0.98	0.98	0.92	0.75	0.87	0.91	1.16	1.01	0.88

## Discussion

Appropriate normative data is essential for the BAFRT in order to be confident about the interpretation of the proportion and valence of statements assigned to any one family member, self, or nobody. To date, no normative data for the BAFRT existed for the Arab community. This study aimed to address this gap in the currently available normative values by measuring responses in a large cohort of Arab children. Additionally, we aimed to provide an assessment of the reliability and validity of the BAFRT in this context.

Test-retest reliability results showed high correlation in BAFRT scores obtained over a 3-week period for both sub-cohorts of children tested. This was true for each of the 27 BART variables and indicated that scores obtained from our cohort of Arab children can be considered as stable over time. We, therefore, proceeded to examine the validity of the test; the first step was to examine convergent validity by assessing the correlation with both the CBCL (father, mother, and teacher) and SDQ tests. All of the CBCL and SDQ scores correlated highly with all BAFRT variables. Negative and dependency categories positively correlated with CBCL and SDQ scores, whereas positive categories negatively correlated with CBCL and SDQ scores. The BAFRT scores were also able to significantly differentiate between cognitively healthy and clinical children, showing discriminant validity of this test in the Arab context. We found that the incoming feelings roughly corresponded to the outgoing feelings in both groups; the main difference was instead found in a greater positivity from the cognitively normal group and greater overall negativity in the statements assigned from the clinical group. Theses test of validity shows the BAFRT is useful for Arab children in settings such as clinical practice in which clinicians may use the test as a means of engaging a child and starting to explore their inner world, rather than just quantitatively examine the scores obtained. As the BAFRT is a simple non-verbal test, this enables many children of all abilities to complete the test and provide useful insights that our results suggest can be considered reliable and valid. While the BAFRT provides a structured way in which to capture family dynamics from a child’s perspective, enabling normative values to be computed for interpretation of the data, the BAFRT also provides much deeper insight into a child’s thought processes and observations that requires an expert to interpret. It is important to be aware that the data can and is often assessed and interpreted at a much deeper level than a simple comparison to normative values. For example, defense mechanisms (avoidance) or lack of attachment maybe two issues that a lack of statements assigned to a family member can expose. Individual cases do need to be evaluated against other evidence to support interpretation and repeat assessments overtime may be needed. Such reasons also highlight the need for large studies when identifying normative values, as only in large numbers can issues like this be diluted.

As the scores obtained from our cohort were deemed reliable and valid, we next sought to calculate appropriate normative values. It has been previously identified that a child’s gender and age impacts upon their feelings toward family members and that this is reflected in the BAFRT scores (Rosen and Brigham, 1984). This is unsurprising as children develop rapidly and change considerably with age. Therefore, we aimed to examine if age and gender effects were present in our cohort and if so, present normative values appropriately. Age was found to have a significant effect on all except one BAFRT variable across the whole cohort and when assessed within the clinical and cognitively healthy groups independently, age was again found to have a significant effect, mainly influencing negative and dependency feelings categories. Gender similarly has a main influence on variables within the negative and dependency feelings categories. It seems that outgoing and incoming positive feelings are more stable over the ages and between genders, but nevertheless, age and gender was found to have a significant overall impact that needed accounting for in the calculation of normative values. In order to present normative values that are useful for further use by researchers and clinicians, we kept our calculations simple. We split our cohort by variables that we found to influence BAFRT scores (age, gender, and clinical status) and simply calculated means and standard deviations for the resulting sub-groups. The resulting values are easy to interpret, replicate and compare.

We acknowledge the limitations of our study; firstly, while a respectable cohort size of 394 was achieved, when we assess the sub-groups of age, gender and clinical status the numbers are reduced greatly. Further work is therefore encouraged to add to and improve upon the accuracy of our normative values, but nevertheless, this sub-grouping was necessary to provide meaningful values and the normative scores presented do still hold value when no others currently exist for Arab children. We also highlight that due to the age of the children in our cohort (5–8) the younger version of the BAFRT was deemed most appropriate to use and our results cannot be compared to those obtained when using the older version of the BAFRT. The younger version is shorter and lacks the strength parameter compared to the older BAFRT, and due to the age of the children the younger version is often administered with more involvement of the examiner (for example, reading out the question). The data presented in this study was obtained from Arab children living in Egypt and Qatar. Therefore, the BAFRT validation and the resulting normative scores are available for immediate reference for family centers in these two countries. However, in order to ensure that the normative scores are equally fit for other Arab cultures more future research should be carried out to examine whether their use is valid for children in other Arab countries such as Saudi Arabia, Behrain, and Yemen. However, we hypothesize that the values obtained in this study are more appropriate for use in other Arab countries than scores obtained in a non-Arabic setting. It is also important to recognize the general limitations of the BAFRT and that these limitations do remain consistent when administered in any language and culture. Issues include; variability in test administration, children not understanding or misinterpreting statements, the unpredictable influence of variable emotions of a child, and the timeframe to which the statements refer to can be unclear and be interpreted differently by each child. All of these limitations impact the reliability, reproducibility and comparison of scores. Many of these issues are common for all childhood psychological measures, often with no practical solutions. Despite the variability, these issues can cause, normative values are still important when used as a guide for a researcher or practitioner and it is crucially important to aim to have these baseline values as accurate as possible.

Overall, we present for the first-time a thorough examination of the reliability and validity of a widely used measure of family relations with Arab children. We conclude that the BAFRT is reliable and valid for use with Arab children and present normative values calculated from our cohort for separate age groups, genders and between clinical and cognitively healthy children. These results provide suitable values for comparison for both further research and clinical practice with Arab children. We emphasize that due to the sample size, the statistical analysis that is conducted provides a thorough and informative examination of the data at this point and that further work is needed in order to determine construct validity of the BAFRT.

## Data Availability Statement

The raw data supporting the conclusions of this article will be made available by the authors, without undue reservation.

## Ethics Statement

The studies involving human participants were reviewed and approved by Department of Psychology, Tanta University, Egypt. Written informed consent to participate in this study was provided by the participants’ legal guardian/next of kin.

## Author Contributions

All authors equally conducted the complete study, including, study design, data acquisition and analysis, and revised the manuscript for intellectual content.

## Conflict of Interest

The authors declare that the research was conducted in the absence of any commercial or financial relationships that could be construed as a potential conflict of interest.

## Abbreviations

BAFRT: Bene-Anthony Family Relations Test
CBCL: child behavior checklist
SDQ: Prosocial Behavior Strengths and Difficulties Questionnaire.
