# Supplementary material for: The Reliability, Validity and Normative Scores of the Bene-Anthony Family Relations Test for Use With Arab Children
Source: Front Psychol. 2021 Jan 27;12:548493. doi: 10.3389/fpsyg.2021.548493 (PMC7873287; doi:10.3389/fpsyg.2021.548493)
Supplement: Supplementary file 1 [file Data_Sheet_1.pdf]

Supplementary Figures and Tables

Supplementary Tables

TableS1: Pearson's Correlation Coefficients of three-week test-retest interval reliability

Variables	r	r^2	Variables	r	r^2
Negative outgoing feelings			Positive outgoing feeling		
Mother	.91**	.83	Mother	.86**	.74
Father	.94**	.88	Father	.92**	.85
Self	.90**	.81	Self	.89**	.79
Sibling	.96**	.92	Sibling	.93**	.86
Friend	.82**	.67	Friend	.91**	.83
Nobody	.90**	.81	Nobody	.88**	.77
Negative incoming feelings			Positive incoming feelings		
Mother	.86**	.74	Mother	.83**	.69
Father	.91**	.83	Father	.89**	.79
Self	.79**	.62	Self	.68**	.46
Sibling	.89**	.79	Sibling	.75**	.56
Friend	.92**	.85	Friend	.82**	.67
Nobody	.86**	.74	Nobody	.81**	.66
Dependency feelings					
Mother	.71**	.50			
Father	.72**	.52			
Sibling	.59**	.35			

** Correlation is significant at the 0.01 level.

TableS2: Replication of three-week test-retest interval reliability; Pearson's Correlation Coefficients

Variables	r	r^2	Variables	r	r^2
Negative outgoing feelings			Positive outgoing feeling		
Mother	.83**	.69	Mother	.87**	.76
Father	.85**	.72	Father	.80**	.64
Self	.79**	.62	Self	.89**	.79
Sibling	.80**	.64	Sibling	.92**	.85
Friend	.80**	.64	Friend	.87**	.76
Nobody	.79**	.62	Nobody	.82**	.67
Negative incoming feelings			Positive incoming feelings		
Mother	.76**	.58	Mother	.88**	.77
Father	.75**	.56	Father	.94**	.88
Self	.78**	.61	Self	.82**	.67
Sibling	.76**	.58	Sibling	.84**	.71
Friend	.87**	.76	Friend	.79**	.62
Nobody	.87**	.76	Nobody	.91**	.83
Dependency feelings					
Mother	.70**	.49			
Father	.80**	.64			
Sibling	.83**	.69			

** Correlation is significant at the 0.01 level.

TableS3: Correlation between all 27 BAFRT variables and the total score of Child Behaviour Checklist (from the father, mother and teacher) and also the Strengths and Difficulties Questionnaire (prosocial behavior- teacher version).

BAFRT	CBCLF		CBCLM		CBCLT		SDQ		BAFRT	CBCLF		CBCLM		CBCLT		SDQ	
Negative outgoing feelings									Positive outgoing feeling								
	r	r^2	r	r^2	r	r^2	r	r^2		r	r^2	r	r^2	r	r^2	r	r^2
Mother	.48**	.23	.49**	.24	.43**	.18	.37**	.14	Mother	-.50**	.25	-.52**	.27	-.48**	.23	-.41**	.17
Father	.45**	.20	.43**	.18	.41**	.17	.39**	.15	Father	-.49**	.24	-.49**	.24	-.43**	.18	-.42**	.18
Self	.49**	.24	.44**	.19	.36**	.13	.40**	.16	Self	-.46**	.21	-.36**	.13	-.48**	.23	-.37**	.14
Sibling	.47**	.22	.43**	.18	.51**	.26	.38**	.14	Sibling	-.51**	.26	-.48**	.23	-.43**	.18	-.38**	.14
Friend	.47**	.22	.43**	.18	.33**	.11	.37**	.14	Friend	-.45**	.20	-.38**	.14	-.43**	.18	-.41**	.17
Nobody	.38**	.14	.45**	.20	.41**	.17	.41**	.17	Nobody	-.50**	.17	-.46**	.21	-.43**	.18	-.42**	.18
Negative incoming feelings									Positive incoming feelings								
Mother	.45**	.20	.45**	.20	.51**	.26	.39**	.15	Mother	-.45**	.20	-.36**	.13	-.49**	.24	-.37**	.14
Father	.41**	.17	.52**	.27	.41**	.17	.41**	.17	Father	-.44**	.19	-.46**	.21	-.42**	.18	-.39**	.15
Self	.35**	.12	.49**	.24	.50**	.25	.37**	.14	Self	-.44**	.19	-.41**	.17	-.45**	.20	-.39**	.15
Sibling	.36**	.13	.48**	.23	.47**	.22	.41**	.17	Sibling	-.46**	.21	-.48**	.23	-.48**	.23	-.39**	.15
Friend	.35**	.12	.48**	.23	.48**	.23	.36**	.13	Friend	-.45**	.20	-.37**	.14	-.45**	.20	-.37**	.14
Nobody	.40**	.16	.44**	.19	.41**	.17	.40**	.16	Nobody	-.45**	.20	-.51**	.26	-.36**	.13	-.41**	.17
Dependency feelings																	

Mother	.37**	.14	.45**	.20	.37**	.14	.38**	.14
Father	.47**	.22	.51**	.26	.38**	.14	.40**	.16
Sibling	.32**	.10	.35**	.12	.31**	.10	.43**	.18

Note: CBCLF = Child Behaviour Checklist Father; CBCLM = Child Behaviour Checklist Mother; CBCLT= Child Behaviour Checklist teacher; SDQ = Strengths and Difficulties Questionnaire (prosocial behaviour- teacher version)

** Correlation is significant at the 0.01 level.

TableS4: Independent sample t test (cognitively healthy compared to clinical group); results for the 27 BAFRT Variables

Variables	*t* (392)	η^2	Variables	*t* (392)	η^2
Negative outgoing feelings			Positive outgoing feeling		
Mother	18.84**	.465	Mother	10.83**	.180
Father	19.43**	.475	Father	13.75**	.198
Self	17.88**	.490	Self	11.51**	.233
Sibling	16.75**	.449	Sibling	10.53**	.262
Friend	16.05**	.317	Friend	9.62**	.304
Nobody	18.92**	.454	Nobody	9.57**	.178
Negative incoming feelings			Positive incoming feelings		
Mother	14.49**	.449	Mother	9.82**	.230
Father	17.22**	.477	Father	10.92**	.325
Self	16.51**	.438	Self	11.79**	.253
Sibling	17.53**	.331	Sibling	13.08**	.220
Friend	15.86**	.410	Friend	9.23**	.191
Nobody	23.38**	.439	Nobody	9.28**	.189
Dependency feelings					
Mother	21.27**	.582			
Father	18.84**	.536			
Sibling	19.43**	.436			

** significant at the 0.01 level.

TableS5: One Way ANOVA results investigating the influence of age group upon BAFRT variable scores across the whole cohort

Variables	F (3, 393)	η^2	Variables	F (3, 393)	η^2

Negative outgoing feelings				Positive outgoing feeling			
	Mother	22.62**	.148		Mother	.576*	.004
	Father	19.44**	.130		Father	3.49*	.026
	Self	22.30**	.146		Self	3.68*	.028
	Sibling	20.65**	.137		Sibling	8.83**	.064
	Friend	24.42**	.158		Friend	6.98**	.051
	Nobody	19.97**	.133		Nobody	3.93*	.029
Negative incoming feelings				Positive incoming feelings			
	Mother	23.91**	.155		Mother	2.74*	.021
	Father	18.78**	.126		Father	5.63**	.041
	Self	22.66**	.148		Self	2.23	-
	Sibling	15.82**	.108		Sibling	6.97**	.051
	Friend	22.44**	.147		Friend	5.43**	.040
	Nobody	23.69**	.154		Nobody	3.69**	.028
Dependency feelings							
	Mother	21.65**	.143				
	Father	17.67**	.120				
	Sibling	16.04**	.110				

** significant at the 0.01 level.

* significant at the 0.05 level.

TableS6: One Way ANOVA for age group in the 27 BAFRT Variables (clinical group only)

Variables		F (3, 121)	η^2	Variables		F (3, 121)	η^2
Negative outgoing feelings				Positive incoming feeling			
	Mother	3.48^{*}	.079		Mother	2.52	-
	Father	1.33	.032		Father	1.10	-
	Self	4.14^{**}	.093		Self	1.13	-
	Sibling	4.32^{**}	.097		Sibling	.51	-
	Friend	14.13^{**}	.259		Friend	.67	-
	Nobody	6.51	.139		Nobody	.90	-
Negative incoming feelings				Positive outgoing feelings			
	Mother	4.17^{**}	.094		Mother	1.71	-
	Father	2.12	.050		Father	.75	-
	Self	5.69^{**}	.124		Self	1.84	-
	Sibling	2.85	.066		Sibling	.48	-
	Friend	8.72^{**}	.178		Friend	.04	-
	Nobody	6.45^{**}	.138		Nobody	.42	-
Dependency feelings							
	Mother	23.37^{**}	.367				
	Father	15.65^{**}	.280				
	Sibling	10.35^{**}	.204				

** significant at the 0.01 level.

* significant at the 0.05 level.

TableS7: One Way ANOVA for age group in the 27 BAFRT Variables (cognitively healthy group only)

Variables		F (3, 265)	η^2	Variables		F (3, 265)	η^2
Negative outgoing feelings		9.71^{**}	.10	Positive outgoing feeling			
	Mother	7.81^{**}	.08		Mother	.13	-
	Father	10.55^{**}	.11		Father	1.35	-
	Self	6.46^{**}	.07		Self	2.04	-
	Sibling	5.95^{**}	.06		Sibling	2.71	-
	Friend	5.16^{**}	.06		Friend	4.35^{**}	-
	Nobody	9.71^{**}	.10		Nobody	1.34	-
Negative incoming feelings				Positive incoming feelings			
	Mother	8.71^{**}	.09		Mother	.61	-
	Father	8.03^{**}	.08		Father	.82	-
	Self	7.92^{**}	.08		Self	.55	-
	Sibling	3.82^{*}	.04		Sibling	2.86	-
	Friend	4.21^{**}	.05		Friend	2.40	-
	Nobody	8.65^{**}	.09		Nobody	1.53	-
Dependency feelings							
	Mother	45.85^{**}	.34				
	Father	32.23^{**}	.27				
	Sibling	15.65^{**}	.15				

** significant at the 0.01 level.

* significant at the 0.05 level.

TableS8: Independent sample t test for BAFRT scores between genders across the whole cohort.

Variables		t (392)	η^2	Variables		t (392)	η^2
Negative outgoing feelings				Positive outgoing feeling			
	Mother	5.65^{**}	.075		Mother	.63	-
	Father	5.87^{**}	.081		Father	1.06	-
	Self	5.97^{**}	.083		Self	.25	-
	Sibling	5.15^{**}	.063		Sibling	.75	-
	Friend	5.46^{**}	.071		Friend	1.00	-
	Nobody	4.69^{**}	.053		Nobody	1.10	-
Negative incoming feelings				Positive incoming feelings			
	Mother	5.55^{**}	.073		Mother	.78	-
	Father	5.07^{**}	.062		Father	.55	-
	Self	5.82^{**}	.080		Self	1.11	-
	Sibling	5.82^{**}	.079		Sibling	1.65	-
	Friend	4.84^{**}	.056		Friend	1.06	-
	Nobody	6.05^{**}	.085		Nobody	.62	-
Dependency feelings							
	Mother	4.01^{**}	.039				
	Father	4.00^{**}	.039				
	Sibling	4.37^{**}	.046				

** significant at the 0.01 level.

TableS9: Independent sample t test between genders for BAFRT scores in clinical children

Variables		t (123)	η^2	Variables		t (123)	η^2
Negative outgoing feelings				Positive outgoing feeling		.42	.00
	Mother	4.06**	.12		Mother	.97	.01
	Father	3.13**	.07		Father	.78	.01
	Self	5.02**	.17		Self	.79	.01
	Sibling	3.61**	.10		Sibling	2.27*	.04
	Friend	2.42*	.05		Friend	.56	.00
	Nobody	5.47**	.20		Nobody	.42	.00
Negative incoming feelings				Positive incoming feelings			
	Mother	4.30**	.13		Mother	1.59	.02
	Father	4.66**	.15		Father	1.28	.01
	Self	5.21**	.18		Self	.68	.00
	Sibling	3.62**	.10		Sibling	.54	.00
	Friend	3.13**	.07		Friend	.18	.00
	Nobody	3.21**	.08		Nobody	.04	.00
Dependency feelings							
	Mother	4.90**	.16				
	Father	4.66**	.15				
	Sibling	2.60*	.05				

** significant at the 0.01 level.

* significant at the 0.05 level.

TableS10: Independent sample t test between genders for BAFRT in cognitively healthy children.

Variables		t (267)	η^2	Variables		t (267)	η^2
Negative outgoing feelings				Positive outgoing feeling		.40	.00
	Mother	6.20^{**}	.13		Mother	.60	.00
	Father	6.23^{**}	.13		Father	.38	.00
	Self	6.00^{**}	.12		Self	.34	.00
	Sibling	6.82^{**}	.15		Sibling	.27	.00
	Friend	5.92^{**}	.12		Friend	.93	.00
	Nobody	6.16^{**}	.12		Nobody	.40	.00
Negative incoming feelings				Positive incoming feelings			
	Mother	6.36^{**}	.13		Mother	.19	.00
	Father	6.65^{**}	.14		Father	.26	.00
	Self	6.63^{**}	.14		Self	.90	.00
	Sibling	5.82^{**}	.11		Sibling	2.45^{*}	.02
	Friend	6.48^{**}	.14		Friend	1.11	.01
	Nobody	5.30^{**}	.10		Nobody	.93	.00
Dependency feelings							
	Mother	4.14^{**}	.06				
	Father	3.82^{**}	.05				
	Sibling	5.05^{**}	.09				

** significant at the 0.01 level.

* significant at the 0.05 level.

Supplementary Figures

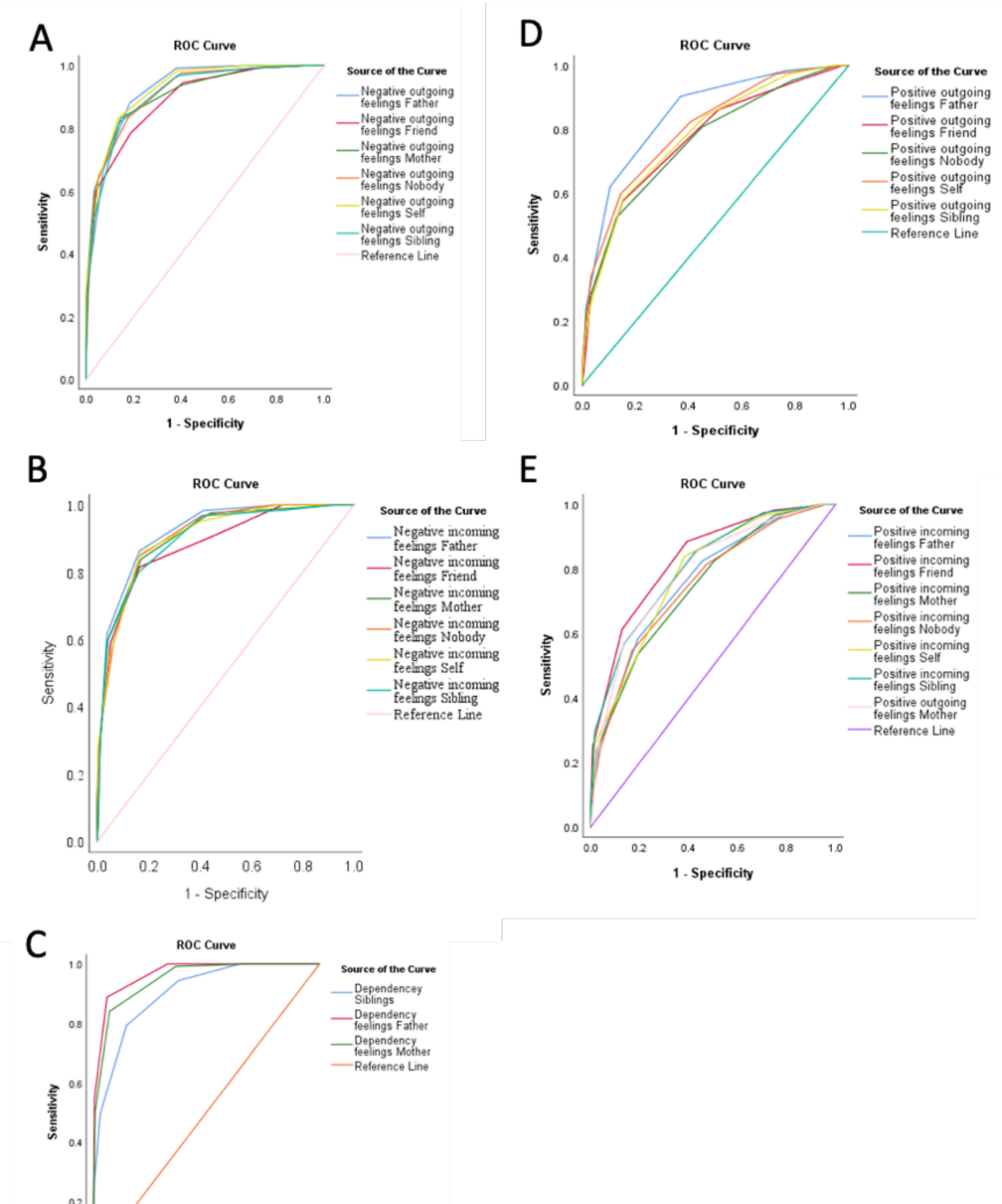
A
ROC Curve
Source of the Curve
Negative outgoing feelings Father
Negative outgoing feelings Friend
Negative outgoing feelings Mother
Negative outgoing feelings Nobody
Negative outgoing feelings Self
Negative outgoing feelings Sibling
Reference Line
Sensitivity
1 - Specificity
B
ROC Curve
Source of the Curve
Negative incoming feelings Father
Negative incoming feelings Friend
Negative incoming feelings Mother
Negative incoming feelings Nobody
Negative incoming feelings Self
Negative incoming feelings Sibling
Reference Line
Sensitivity
1 - Specificity
C
ROC Curve
Source of the Curve
Dependencey Siblings
Dependency feelings Father
Dependency feelings Mother
Reference Line
Sensitivity
D
ROC Curve
Source of the Curve
Positive outgoing feelings Father
Positive outgoing feelings Friend
Positive outgoing feelings Nobody
Positive outgoing feelings Self
Positive outgoing feelings Sibling
Reference Line
Sensitivity
1 - Specificity
E
ROC Curve
Source of the Curve
Positive incoming feelings Father
Positive incoming feelings Friend
Positive incoming feelings Mother
Positive incoming feelings Nobody
Positive incoming feelings Self
Positive incoming feelings Sibling
Positive outgoing feelings Mother
Reference Line
Sensitivity
1 - Specificity

FigS1: ROC curves displaying the performance of the BAFRT variables for the discrimination of clinical and cognitively normal children. A = Negative outgoing feelings. B = Negative incoming feelings. C = Dependency Feelings. D = Positive outgoing feelings. E = Positive incoming feelings.
